# A zebrafish larval model reveals early tissue-specific innate immune responses to *Mucor circinelloides*

**DOI:** 10.1242/dmm.019992

**Published:** 2015-11-01

**Authors:** Kerstin Voelz, Remi L. Gratacap, Robert T. Wheeler

**Affiliations:** 1 Institute of Microbiology and Infection, School of Biosciences, University of Birmingham B15 2TT, UK; 2 Department of Molecular and Biomedical Sciences, University of Maine, Orono, ME 04469, USA; 3 National Institute of Health Research Surgical Reconstruction and Microbiology Research Centre, Queen Elizabeth Hospital, Birmingham B15 2TH, UK

**Keywords:** Zebrafish larva, *Danio rerio*, Mucormycosis, Innate immune system, Phagocytes, Macrophages, Neutrophils, *Mucor circinelloides*

## Abstract

Mucormycosis is an emerging fungal infection that is clinically difficult to manage, with increasing incidence and extremely high mortality rates. Individuals with diabetes, suppressed immunity or traumatic injury are at increased risk of developing disease. These individuals often present with defects in phagocytic effector cell function. Research using mammalian models and phagocytic effector cell lines has attempted to decipher the importance of the innate immune system in host defence against mucormycosis. However, these model systems have not been satisfactory for direct analysis of the interaction between innate immune effector cells and infectious sporangiospores *in vivo*. Here, we report the first real-time *in vivo* analysis of the early innate immune response to mucormycete infection using a whole-animal zebrafish larval model system. We identified differential host susceptibility, dependent on the site of infection (hindbrain ventricle and swim bladder), as well as differential functions of the two major phagocyte effector cell types in response to viable and non-viable spores. Larval susceptibility to mucormycete spore infection was increased upon immunosuppressant treatment. We showed for the first time that macrophages and neutrophils were readily recruited *in vivo* to the site of infection in an intact host and that spore phagocytosis can be observed in real-time *in vivo*. While exploring innate immune effector recruitment dynamics, we discovered the formation of phagocyte clusters in response to fungal spores that potentially play a role in fungal spore dissemination. Spores failed to activate pro-inflammatory gene expression by 6 h post-infection in both infection models. After 24 h, induction of a pro-inflammatory response was observed only in hindbrain ventricle infections. Only a weak pro-inflammatory response was initiated after spore injection into the swim bladder during the same time frame. In the future, the zebrafish larva as a live whole-animal model system will contribute greatly to the study of molecular mechanisms involved in the interaction of the host innate immune system with fungal spores during mucormycosis.

## Abstract (precis)

**Summary:** We use zebrafish larval models to delineate the temporospatial patterns of innate immune responses to fungal infections with mucormycete spores.

## INTRODUCTION

Mucormycosis is a fungal infection with extremely high mortality rates (30-90%; Brown et al., 2012a). Disease is caused by a spectrum of species belonging to the Mucorales (e.g. *Rhizopus oryzae*, *Mucor circinelloides*, *Leichtheimia corymbifera*). Although mucormycosis has historically been considered a rare disease, advances in medical care and an ageing population have resulted in a recent increase in the incidence of this fungal infection (Roden et al., 2005; Chayakulkeeree et al., 2006; Roilides et al., 2009; Kontoyiannis et al., 2010; Petrikkos et al., 2012), such that mucormycosis is now the second most prevalent mould infection (Brown et al., 2012b). Recent data from France show an increase of 7.3% in the incidence and 9.3% in the mortality rate of mucormycosis cases between 2001 and 2010 (Bitar et al., 2014), with one of the causative species, *R. oryzae*, now being responsible for an estimated >10,000 cases per year worldwide (Brown et al., 2012b). Treatment of mucormycosis is very costly, with an average expense of $100,000 per case (Ibrahim et al., 2009), but remains unsuccessful in most individuals. In addition, reports of mucormycosis outbreaks have become increasingly frequent in recent years. In September 2013, 200 individuals in the USA presented with foodborne mucormycosis (Lee et al., 2014); 13 mucormycosis cases occurred after a tornado in Joplin in 2011 (Neblett Fanfair et al., 2012); and 16 cases of invasive mould infection by Mucorales following combat-related injuries were reported in American soldiers in 2010 (Warkentien et al., 2012).

Mucormycosis most often presents as rhinocerebral, sinal, soft tissue, skin, gastrointestinal or disseminated disease (Mendoza et al., 2014). Symptoms are caused by germination of asexual spores (sporangiospores) within susceptible individuals, leading to severe angioinvasion by hyphal structures, causing thrombosis and tissue necrosis (Ibrahim et al., 2005; Ben-Ami et al., 2009). Current antifungal therapy is ineffective, and treatment involves extensive surgical removal of infected tissue, often leading to limb amputation and long-term disability. Susceptible individuals often present with immune defects, such as neutropenia and impaired macrophage or neutrophil functions owing to corticosteroid therapy or tissue trauma (Ribes et al., 2000; Chayakulkeeree et al., 2006; Ibrahim et al., 2012; Petrikkos et al., 2012).

Early research studies demonstrated the importance of the innate immune system and, in particular, phagocytic effector cells in controlling mucormycete infections. Histological studies in a rabbit model linked a lack of early recruitment of phagocytes to the site of mucormycete infection in diabetic hosts to invasive and fatal disease (Sheldon and Bauer, 1959). Murine bronchoalveolar macrophages inhibit germination of *R. oryzae* spores and thus prevent formation of invasive hyphae *in vitro* (Waldorf et al., 1984a; Levitz et al., 1986; Waldorf, 1989; Jorens et al., 1995). However, macrophages are unable to kill fungal spores (Levitz et al., 1986; Waldorf, 1989). In addition, macrophages from diabetic or corticosteroid-treated mice fail to inhibit spore germination (Waldorf et al., 1984a,b). Infectious spores do not possess chemotactic potential to induce neutrophil migration (Waldorf and Diamond, 1985) and are resistant to neutrophil killing *in vitro* (Levitz et al., 1986). Conversely, swollen spores and hyphal structures are sensitive to neutrophil-mediated damage by oxidative and cationic peptides *in vitro* (Diamond and Clark, 1982; Levitz et al., 1986). However, this hyphal damage is relatively low compared with the damage that human polymorphonuclear neutrophils can exert on other filamentous fungi, i.e. *Aspergillus fumigatus* (Chamilos et al., 2008a). Host expression studies of *R. oryzae* infections in the fruit fly infection model showed downregulation of genes in several pathways, including pathogen recognition, immune defence and stress responses (Chamilos et al., 2008b).

**TRANSLATIONAL IMPACT**

**Clinical issue**

Invasive fungal infections have become a significant health risk and burden on health-care systems over the past decade. Even previously rare infections are now emerging more frequently, owing to an increase in susceptible individuals. Mucormycosis is one such fungal infection, but its disease mechanisms are greatly understudied. In addition, current antifungal therapies are insufficient and infections often fatal. Previous observations of mucormycosis in mammalian models showed the importance of our innate immune system to fight this infection. However, these traditional model systems do not allow direct studies of the interaction between infectious fungal spores and innate immune cells.

**Results**

The authors used zebrafish larvae infected with spores from the fungus *Mucor circinelloides* to investigate these host-pathogen interactions in real time by high-resolution microscopy. This model shows extensive similarity to the human disease; for example, depending on the site of infection (the hindbrain ventricle or the swim bladder), the authors reported differences in disease severity, resembling the site-specific susceptibility to infection typical of the human clinical presentation. Disease severity is also correlated with differences in immune responses, particularly in the recruitment of phagocytes and the expression of immune signalling molecules (cytokines).

**Implications and further directions**

This zebrafish larval model enables *in vivo* investigations of fungal spore interactions with innate immune effector cells (phagocytes) by high-resolution real-time microscopy. In the future, this whole-animal model system will greatly contribute to the study of molecular mechanisms involved in the interaction between the host innate immune system and fungal spores during mucormycosis.

Although all these observations clearly demonstrate the importance of phagocytes in the innate immune response to mucormycosis, the molecular and cellular nature of their interaction is less well defined. The lack of fungicidal activity of macrophages together with fungal dissemination raises questions about the importance of macrophages in inhibiting spore germination. The literature suggests a protective effect of immediate phagocyte recruitment to the site of infection (Sheldon and Bauer, 1959).

Our current understanding of the pathogenesis of mucormycosis is based on *in vitro* analysis of mucormycete interactions with isolated mammalian cells and end-point assays in rodent models. The former is a highly malleable experimental system but lacks direct insights into disease progression. By contrast, disease aetiology can be studied within animal hosts, but little detailed information on cellular processes involved can be obtained. Hence, these models are not suitable to address the key questions about temporospatial patterns of the innate immune response to infections with mucormycete spores that have been unanswered for several decades.

In recent years, the zebrafish (*Danio rerio*) larval model has become an accepted model system for the study of infectious disease pathogenesis (Neely et al., 2002; Prouty et al., 2003; van der Sar et al., 2004; Sullivan and Kim, 2008; Li and Hu, 2012; Tobin et al., 2012) that offers several advantages over rodent animal hosts. Its transparency and small size allows for microscopic real-time studies of host-pathogen interactions on a cellular level across the whole animal. In addition, the zebrafish innate immune system is similar to the human (Brothers et al., 2011; Brothers and Wheeler, 2012; Tobin et al., 2012), and early larval stages do not have an adaptive response, thereby enabling a more focused analysis of innate immune responses. Elegant experimental approaches (i.e. microinjection) allow for tissue-targeted infection, enabling powerful analysis of tissue-specific responses to pathogens (Tobin et al., 2012).

In this study, we have used the zebrafish (*D. rerio*) larva as an *in vivo* model system to enable an integrative approach to study the pathogenesis of mucormycosis. Initially, we established the zebrafish larval model by demonstrating that it mimics a range of aspects of human mucormycosis. We showed differential virulence in two infection sites. In addition, immunosuppressant treatment increased larval susceptibility to mucormycete spore infection. We then applied this model system to investigate the temporospatial events of the early innate immune response to mucormycete spores. Macrophages and neutrophils were readily recruited to the site of infection, and spore phagocytosis was observed in real time *in vivo*. In addition, viable and non-viable spores failed substantially to activate pro-inflammatory gene expression by 6 h post-infection (h.p.i.) in both infection models. After 24 h, induction of a pro-inflammatory response was predominantly observed in hindbrain ventricle infections with viable spores but not swim bladder infections. Taken together, we addressed the previously reported *in vitro* lack of chemotactic potential of infectious spores *in vivo* and gained a better understanding of early events of a failed immune response leading to fatal mucormycosis as well as some mechanistic insight into disease dissemination.

## RESULTS

### Zebrafish larvae are susceptible to *M. circinelloides* NRRL3631 in a hindbrain infection model

Infection with mucormycete spores most often occurs through inhalation, ingestion or forceful mechanical deposition (e.g. natural disasters, explosive devices). After initial infection, rhinocerebral, cerebral and disseminated disease, characterized by invasive fungal structures, are the most common presentations of mucormycosis (Dromer and McGinnis, 2003; Ibrahim and Spellberg, 2006). We evaluated infection in AB wild-type zebrafish larvae by microinjecting fungal spores into the hindbrain ventricle, an infection system previously described as a model of disseminated candidiasis (Brothers et al., 2011) and invasive aspergillosis (Knox et al., 2014; Herbst et al., 2015).

**Fig. 1.**
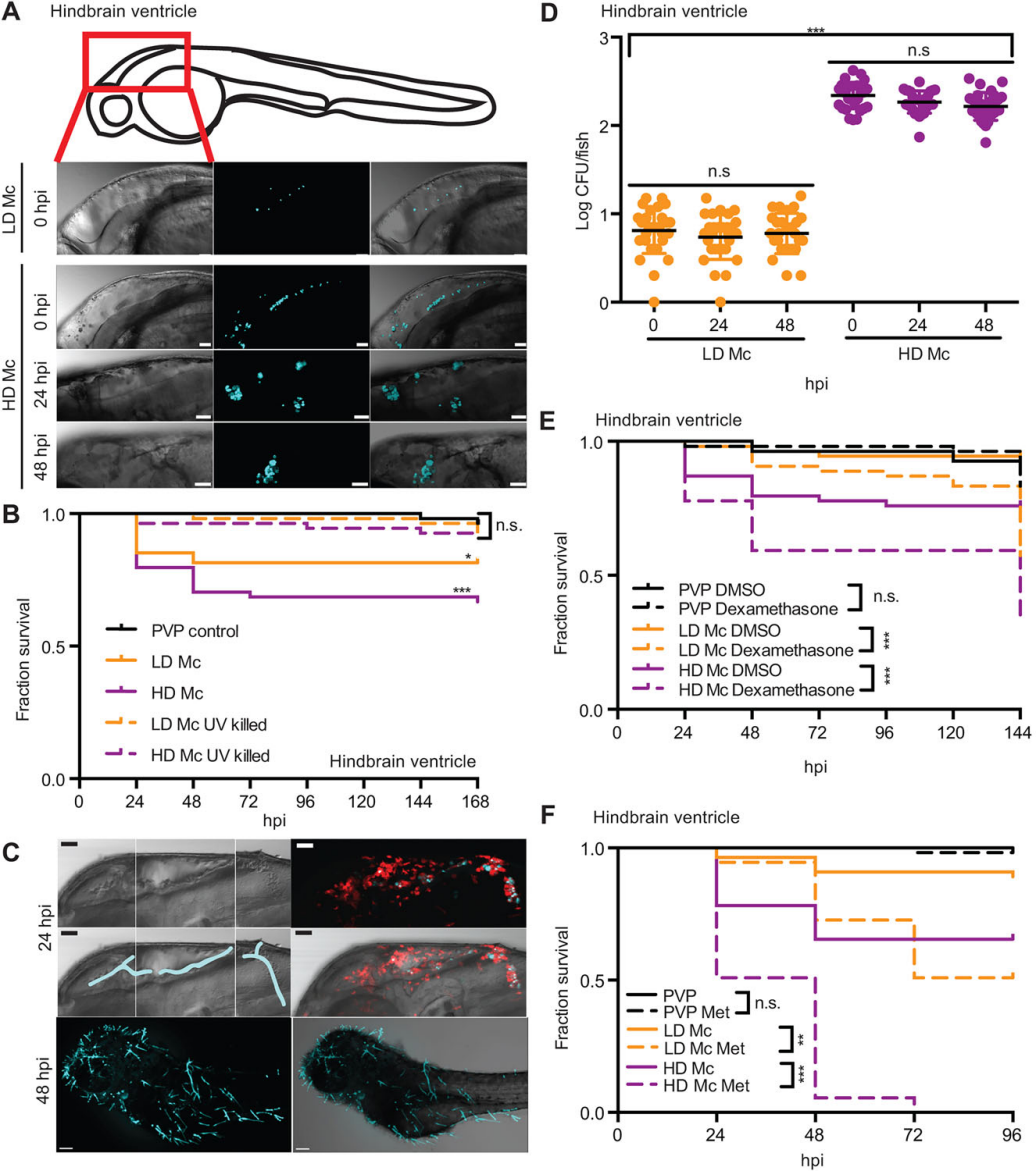
**Zebrafish larvae are susceptible to the mucormycete *M. circinelloides* NRRL3631 in a hindbrain infection model.** (A) AB wild-type zebrafish larva were injected in the hindbrain ventricle at prim-25 stage with a low dose (LD Mc, approximately 10 spores) or a high dose of *Mucor* *circinelloides* NRRL3631 (HD Mc, approximately 100 spores) and monitored over time (representative *z*-stacks: 28, 27, 23 and 40 sections every 3 μm, respectively; scale bar: 40 μm). (B) Injection of LD and HD Mc mucormycete spores into the hindbrain ventricle of prim-25 larvae induced significant mortality. Hindbrain ventricle infection with UV-killed spores did not cause significant mortality in AB wild-type larvae compared with the PVP control. (C) Hyphal growth invading the forebrain and ventral muscular layers from posterior hindbrain (representative images of HD Mc infection; top and middle right: DIC montage with filamentous growth highlighted in blue; fluorescent *z*-stacks: 61 sections every 4.4 μm; scale bar: 50 μm, upper two panels). Dead fish, previously injected with viable spores, presented with filamentous fungal growth (representative image from LD Mc hindbrain injection; cyan, spores/hyphae; red, macrophages; scale bar: 100 μm). (D) Spores remained viable within the zebrafish larvae over the time course of 48 h post-infection (h.p.i.). (E,F) Larval immunosuppression increases susceptibility to infections with *M. circinelloides* NRRL3631. (E) AB wild-type zebrafish larvae treated with dexamethasone after hindbrain ventricle injection showed significantly increased mortality at the low as well as the high infection dose compared with DMSO-treated infected larvae. (F) In comparison to untreated infected *Tg(mpeg1:G/U:NfsB-mCherry)* larvae, larval treatment with metronidazole (Met) significantly increased susceptibility to low- and high-dose hindbrain ventricle infection with fungal spores.

Infections of zebrafish larvae in the hindbrain ventricle at the prim-25 stage (∼36 h post-fertilization; Kimmel et al., 1995) were conducted with the clinical isolate *M. circinelloides* NRRL3631 at a low or a high injection dose (10^7^ and 10^8^ spores/ml, respectively; Fig. 1A). This isolate has been described to produce homogeneously small spores, allowing for experimentally consistent injections. In addition, this strain also exhibits a normal developmental cycle (as opposed to the large spore isolate CBS277.49 used later in this study; Li et al., 2011). Significant mortality was observed for low and high injection doses (17.9 and 28.4%, respectively) compared with the polyvinylpyrrolidone 40 (PVP) vehicle control [PVP versus low-dose *M. circinelloides* (LD Mc) *P*<0.01; PVP versus high-dose *M. circinelloides* (HD Mc) *P*<0.001; low versus high dose *P*=0.09, *n*=54] when spores were injected into the hindbrain ventricle (Fig. 1B).

Taken together, the data demonstrate that zebrafish larvae are susceptible to the mucormycete *M. circinelloides* after infection of the hindbrain ventricle.

### *M. circinelloides* NRRL3631 virulence is associated with viable spores forming invasive hyphae in the hindbrain infection model

Mortality can be caused either by direct effects (e.g. toxin production or invasive hyphal growth) exerted by a pathogen or by indirect effects, such as overstimulation of the immune system leading to excessive inflammation. To test the mode of mortality observed after hindbrain ventricle infection, we conducted survival studies with ultraviolet (UV)-killed spores.

Infection with UV-killed spores did not cause significant mortality compared with the PVP control in AB wild-type larvae (PVP versus LD Mc UV killed *P*=0.400; PVP versus HD Mc UV killed *P*=0.389, *n*=54; Fig. 1B). After injection of viable spores, filamentous fungal growth was found in all dead fish (independent of infection dose; Fig. 1C, lower panel). We attempted to visualize the fungal growth leading to larval death and observed invasive fungal growth. Filaments from the hindbrain reached into the forebrain and invaded the ventral muscular tissue from the posterior end of the hindbrain (Fig. 1C, upper panels). Interestingly, independent of the outcome of infection, spores remained viable within the zebrafish larvae over the time course of 48 h.p.i., with no significant differences in colony-forming units (CFU) over time (*n*=30, pooled data from three independent experimental repeats with *n*=10 each; Fig. 1D).

In summary, mortality in the hindbrain infection model is associated with invasive hyphal growth of viable fungal spores. Spores were not cleared in infected larvae up to 48 h.p.i.

### Larval immunosuppression increases susceptibility to infections with *M. circinelloides* NRRL3631 in the hindbrain infection model

Immunosuppression and impaired phagocyte function resulting from corticosteroid treatment, uncontrolled diabetes or trauma are major predeposing factors for mucormycosis (Mendoza et al., 2014). We investigated the effect of treatment with the corticosteroid dexamethasone (10 μg/ml), which induces general immunosuppressant effects in AB wild-type zebrafish larvae.

AB wild-type zebrafish larvae treated with dexamethasone after hindbrain ventricle injection showed significantly increased mortality at low as well as high infection dose (42.6 and 64.8%, respectively) compared with dimethyl sulfoxide (DMSO)-treated infected larvae (*P*<0.001, *n*=54; Fig. 1E). Dexamethasone alone did not significantly increase larval mortality compared with DMSO-treated controls until 144 h.p.i. (*P*=0.170; Fig. 1E). We did not observe significant mortality levels with low-dose infection compared with the DMSO-treated PVP control (*P*=0.717). We believe this is because treatment with DMSO induced some larval mortality in the PVP DMSO control (8%), which is normally very low in the PVP control (0-4%).

In addition, macrophages were specifically depleted by treatment of *Tg(mpeg1:G/U:NfsB-mCherry)* larvae with the prodrug metronidazole (10 μg/ml), which is processed to its cytotoxic form by an *nfsb*-encoded nitroreductase. Successful depletion was assured by counting of mCherry^+^ cells in the caudal haematopoetic tissue, six somites posterior to the anal vent (Fig. S1A,B). In comparison to untreated infected larvae, larval treatment with metronidazole also significantly increased mortality in response to low- and high-dose hindbrain ventricle infection with fungal spores (49.1 and 100%, respectively; LD Mc versus LD Mc metronidazole *P*=0.003, HD Mc versus HD Mc metronidazole *P*<0.001, *n*=55; Fig. 1F). Metronidazole treatment alone did not significantly alter larval viability up to 96 h.p.i. in the hindbrain ventricle (PVP versus PVP metronidazole *P*=0.317; Fig. 1F). Low-dose injections resulted in significant larval mortality compared with the PVP control (*P*=0.012).

In conclusion, both immunomodulatory approaches resulted in significantly increased mortality in the zebrafish larvae hindbrain ventricle infection model. In particular, macrophage depletion dramatically increased larval susceptibility to mucormycete spores when microinjected into the hindbrain ventricle, suggesting that macrophages are essential in the host response to fungal spores in this infection model.

### Phagocytes are recruited to the site of injection in the hindbrain infection model

Our macrophage ablation study and previous research have indicated the importance of phagocytes in mounting a protective immune response to mucormycete spores (Waldorf et al., 1984a,b). Macrophages are important in controlling mucormycosis by inhibiting fungal spore germination in the healthy host (Waldorf et al., 1984a; Levitz et al., 1986; Waldorf, 1989; Jorens et al., 1995), and a lack of chemotactic neutrophil response and ability to kill infectious spores has been reported previously *in vitro* (Waldorf and Diamond, 1985; Levitz et al., 1986). We investigated the dynamics of recruitment of phagocytes using two transgenic zebrafish lines with fluorescently labelled macrophages and neutrophils. *Tg(mpeg1:G/U:NfsB-mCherry)* (red-fluorescent macrophages) and *Tg(mpx:GFP)* (green-fluorescent neutrophils) zebrafish larvae were independently injected with a low or high dose of mucormycete spores to observe recruitment of phagocytes to the site of infection at 4 and 24 h.p.i.

**Fig. 2.**
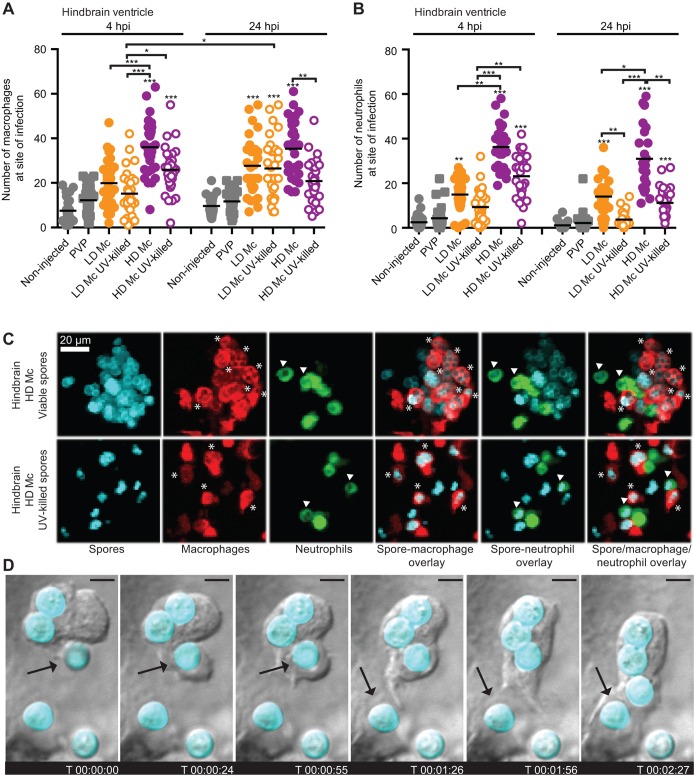
**Phagocytes are recruited to the site of infection after spore injection into the hindbrain and interact with spores *in vivo*.** Macrophage (A) and neutrophil recruitment (B) to the site of spore injection in the hindbrain ventricle of *Tg(mpeg1:G/U:NfsB-mCherry)* and *Tg(mpx:GFP)* zebrafish larvae, respectively, was observed at 4 and 24 h.p.i. Pooled data presented were obtained from three independent experimental repeats with 10 larvae each. (C) Spores can be observed inside macrophages (indicated by asterisks) and neutrophils (indicated by arrowheads) after injection of viable and non-viable spores in the hindbrain ventricle of *Tg(mpeg1:G/U:NfsB-mCherry/mpx:GFP)* larvae (representative images at 10 h 45 min and 1 h 5 min, respectively; *z*-stack: 15 sections every 7.3 μm; scale bar: 20 μm). (D) Several subsequent events of spore phagocytosis were also observed in an undefined phagocyte *in vivo* (arrow indicates next spore to be phagocytosed; time is expressed as h:min:s; scale bar: 4 μm; see also Movie 1).

Significantly higher numbers of macrophages were found at the site of infection 4 h after injection of a high dose of viable or non-viable spores compared with the PVP control injections (*P*<0.001) in the hindbrain ventricle. No significant macrophage recruitment was seen at this time after injection of a low dose of viable or non-viable spores. Macrophage recruitment after high-dose challenge with viable or non-viable spores was also significantly higher than after low-dose challenge with viable or non-viable spores, respectively (LD Mc versus HD Mc viable *P*<0.001; LD Mc versus HD Mc non-viable *P*<0.05). Injection of non-viable spores showed less macrophage recruitment than injection with viable spores in the low- and high-dose challenges, although no statistically significant difference was detected at 4 h.p.i. (Fig. 2A and Table S1A). At 24 h.p.i., significantly higher numbers of macrophages were observed in the hindbrain in larvae injected with low-dose viable, low-dose non-viable and high-dose viable spores compared with PVP control injections (*P*<0.001). No statistically significant difference in macrophage numbers at the injection site was detected between low-dose and high-dose injections of viable or non-viable spores at this time point; however, significantly fewer macrophages were seen at the site of injection in larvae challenged with a high dose of non-viable spores compared with larvae challenged with a high dose of viable spores (*P*<0.01) at 24 h.p.i. (Fig. 2A and Table S1A).

Significantly higher numbers of neutrophils were found at the site of infection 4 h after injection of a low or high dose of viable or a high dose of non-viable spores compared with the PVP control injections (*P*<0.001) into the hindbrain ventricle. No significant neutrophil recruitment was seen at this time point after injection of a low dose of non-viable spores. Neutrophil recruitment after high-dose challenge with viable or non-viable spores was also significantly higher than after low-dose challenge with viable or non-viable spores, respectively (low- versus high-dose viable *P*<0.01; low- versus high-dose non-viable *P*<0.01). Injection of non-viable spores provoked less neutrophil recruitment than injection with viable spores at low- and high-dose challenge, although no statistically significant difference was detected at 4 h.p.i. (Fig. 2B and Table S1B). At 24 h.p.i., significantly higher numbers of neutrophils were observed in the hindbrain in larvae injected with low-dose viable, high-dose viable and high-dose non-viable spores compared with PVP control injections (*P*<0.001). An increased number of neutrophils at the site of injection in larvae challenged with a high compared with a low dose of viable spores remained at 24 h.p.i. (*P*<0.05). Significantly lower numbers of neutrophils at the injection site were detected in larvae injected with non-viable spores compared with larvae injected with viable spores (LD Mc versus HD Mc non-viable *P*<0.01; HD Mc viable versus HD Mc non-viable *P*<0.01; Fig. 2B and Table S1B).

Viable and non-viable spores were observed inside macrophages and neutrophils, indicating that they had been phagocytosed (Fig. 2C). We also observed spore uptake by a phagocytic cell in real time (Fig. 2D and Movie 1).

In summary, in contrast to previous *in vitro* studies, macrophages as well as neutrophils are recruited in a dose-dependent manner to the site of spore infection in the hindbrain ventricle zebrafish larval model of mucormycosis at 4 and 24 h.p.i. UV-killed spores resulted in reduced neutrophil recruitment in hindbrain injections after low- and high-dose challenge, whereas a similar observation was made only for macrophage recruitment after high-dose injections. This suggests that these two innate immune cells respond in a different manner to hindbrain ventricle infection with live and dead pathogen over time, and indicates that differential activation and chemotactic recruitment mechanisms are active during phagocyte recruitment.

### Phagocytes accumulate at the site of live spore injection in the hindbrain infection model

**Fig. 3.**
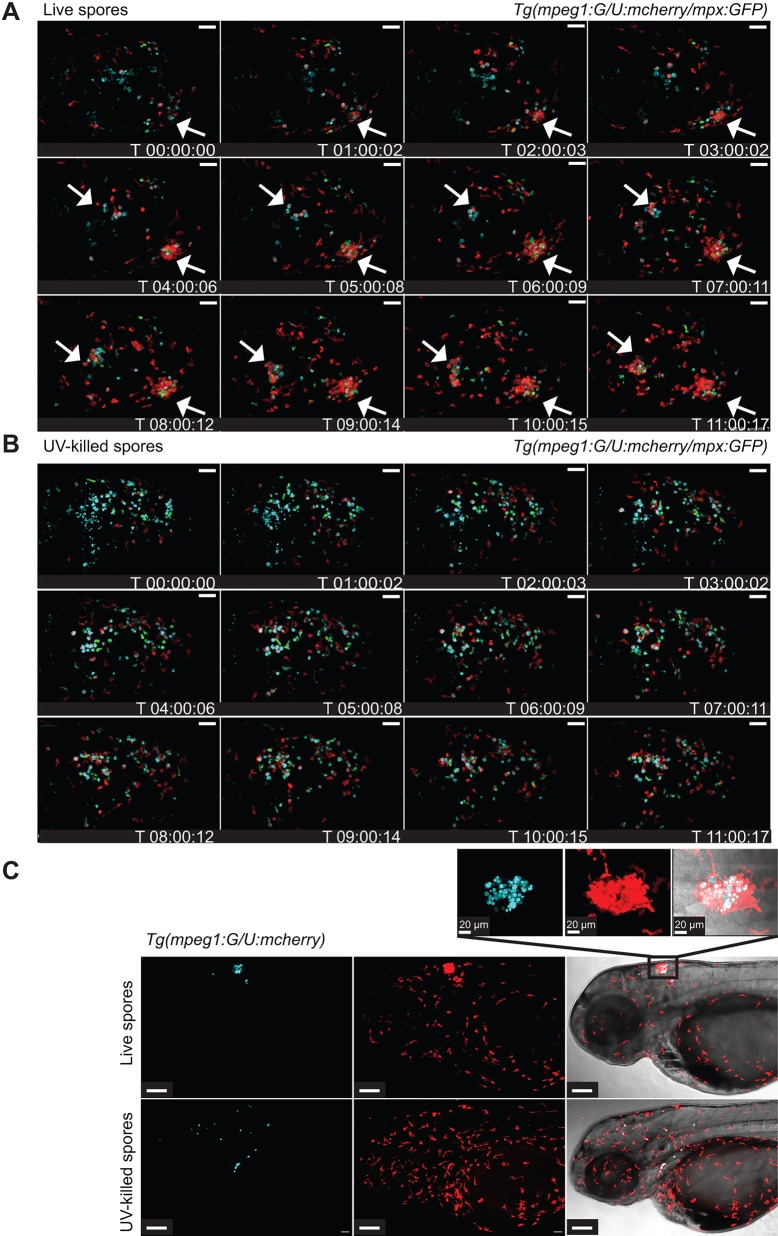
**Phagocytes accumulate at the site of live spore injection in the hindbrain infection model.** (A) Macrophages and neutrophils accumulate around viable spores (*z*-stack: 15 sections every 7.3 μm; scale bar: 40 μm; see also Movie 2). (B) Significantly less macrophage and neutrophil clustering was observed in larvae injected with UV-killed spores (*z*-stack: 15 sections every 7.3 μm; scale bar: 40 μm; see also Movie 3). (C) Formation of clusters around viable spores was correlated with low levels of spore dissemination, whereas non-viable spores did disseminate efficiently throughout the fish (*z*-stacks: 30 sections every 10.35 and 9.25 μm, respectively, scale bars: 50 μm; zoomed in section: *z*-stack 31 sections every 1 μm). Time is expressed as h:min:s starting at 1 h after injection.

During our analysis of phagocyte recruitment, we observed an intriguing phenotype of phagocytes clustering around live spores at the site of infection (hindbrain ventricle) in dense structures. Interestingly, similar dense immune cell clusters have previously been described in fungal infections (Dubey et al., 2005), although the function of the clusters is currently unknown. Hence, we performed real-time confocal microscopy to observe phagocyte recruitment in *Tg(mpeg1:G/U:NfsB-mCherry/mpx:GFP)* zebrafish larvae. Macrophages and neutrophils showed clustering around viable spores (Fig. 3A, Table 1 and Movie 2). Although this movement appeared to be directed and clusters appeared to be stable on visual inspection, we cannot currently exclude the possibility of random or anchored leucocyte movement or chemotaxis and the possibility of phagocytes slowing down to allow spore phagocytosis before moving away. However, injection of non-viable spores into the hindbrain of *Tg(mpeg1:G/U:NfsB-mCherry/mpx:GFP)* larvae did not show a similar cluster formation (Fig. 3B and Movie 3). We quantified cluster formation in two transgenic zebrafish lines independently; *Tg(mpeg1:G/U:NfsB-mCherry)* and *Tg(mpx:GFP)*. Significantly fewer zebrafish larvae showed macrophage (LD Mc versus LD Mc UV killed *P*<0.001; HD Mc versus HD Mc UV killed *P*<0.001) or neutrophil accumulation (LD Mc versus LD Mc UV killed *P*=1.0; HD Mc versus HD Mc UV killed *P*<0.001) after injection of UV-killed spores compared with injection of live spores (Fig. 3C and Table 1). Interestingly, the formation of clusters around viable spores was correlated with low levels of spore dissemination (defined as observation of at least one spore not localized at the initial site of injection), whereas non-viable spores disseminated significantly more often in larvae injected with non-viable spores compared with larvae injected with viable spores [Fig. 3C and Table 1; *Tg(mpeg1:G/U:NfsB-mCherry)*: LD Mc versus LD Mc UV killed *P*=0.056; HD Mc versus HD Mc UV killed *P*<0.001; *Tg(mpx:GFP)*: LD Mc versus LD Mc UV killed *P*=0.012; HD Mc versus HD Mc UV killed *P*<0.001].

**Table 1.**
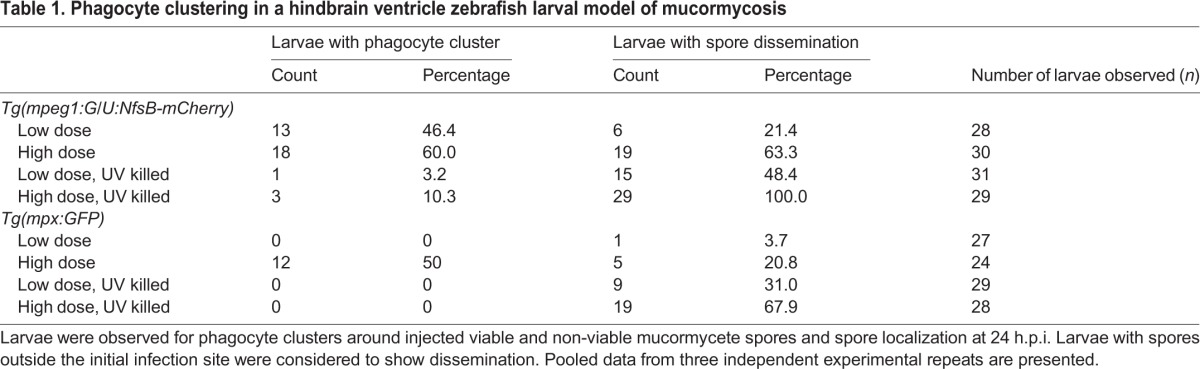
**Phagocyte clustering in a hindbrain ventricle zebrafish larval model of mucormycosis**

These observations highlight a differential phagocyte response to viable and non-viable spores.

### Mucormycete spores induce differential pro-inflammatory cytokine responses dependent on spore viability in the hindbrain infection model

Cytokine signalling is involved in phagocyte recruitment and mounting of the protective pro-inflammatory response to pathogens. Therefore, we investigated mRNA expression of the pro-inflammatory mediators *il1b* and *tnfa* in response to viable and non-viable mucormycete spores at 6 and 24 h.p.i. by quantitative real-time PCR.

**Fig. 4.**
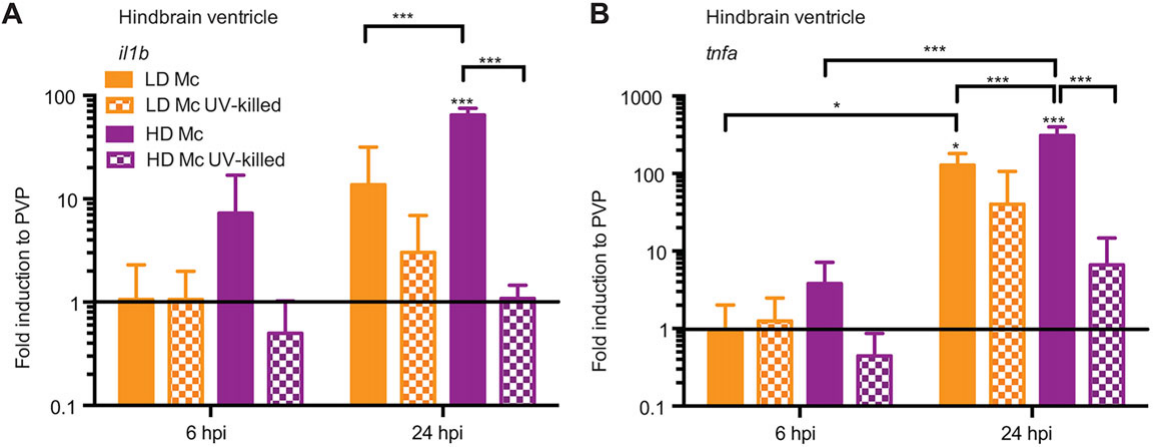
**Mucormycete spores induce differential pro-inflammatory cytokine expression dependent on spore viability in the hindbrain infection model.** Initial mRNA levels for both cytokines were low at 6 h.p.i. (A) but increased after 24 h.p.i. (B) for infections with live spores. At 24 h.p.i., high-dose infection with viable spores induced significantly higher mRNA levels than infection with a low dose of viable spores and non-viable spores. Infection with non-viable spores did not induce significant cytokine expression at either time point.

Initial mRNA levels for both cytokines were low at 6 h.p.i. in the hindbrain ventricle model, with no significant expression levels compared with the PVP control. At 24 h.p.i., significant levels of *il1b* were transcribed after a high-dose viable spore challenge (*P*<0.001) and of *tnfa* after low- and high-dose injection of viable spores (*P*<0.05 and *P*<0.001, respectively; Fig. 4A,B). High-dose infection with viable spores induced higher mRNA levels than infection with a low dose of viable spores (*P*<0.001, *n*=3), and cytokine induction after high-dose challenge with non-viable spores was significantly lower than after high-dose viable spore challenge (*P*<0.001; Fig. 4A,B).

Taken together, mucormycete spores elicit a pro-inflammatory cytokine response at 24 h.p.i. in the zebrafish larval hindbrain ventricle, with a differential response to viable and non-viable spores as well as infection dose.

### Zebrafish larval responses to *M. circinelloides* in a swim bladder infection model

**Fig. 5.**
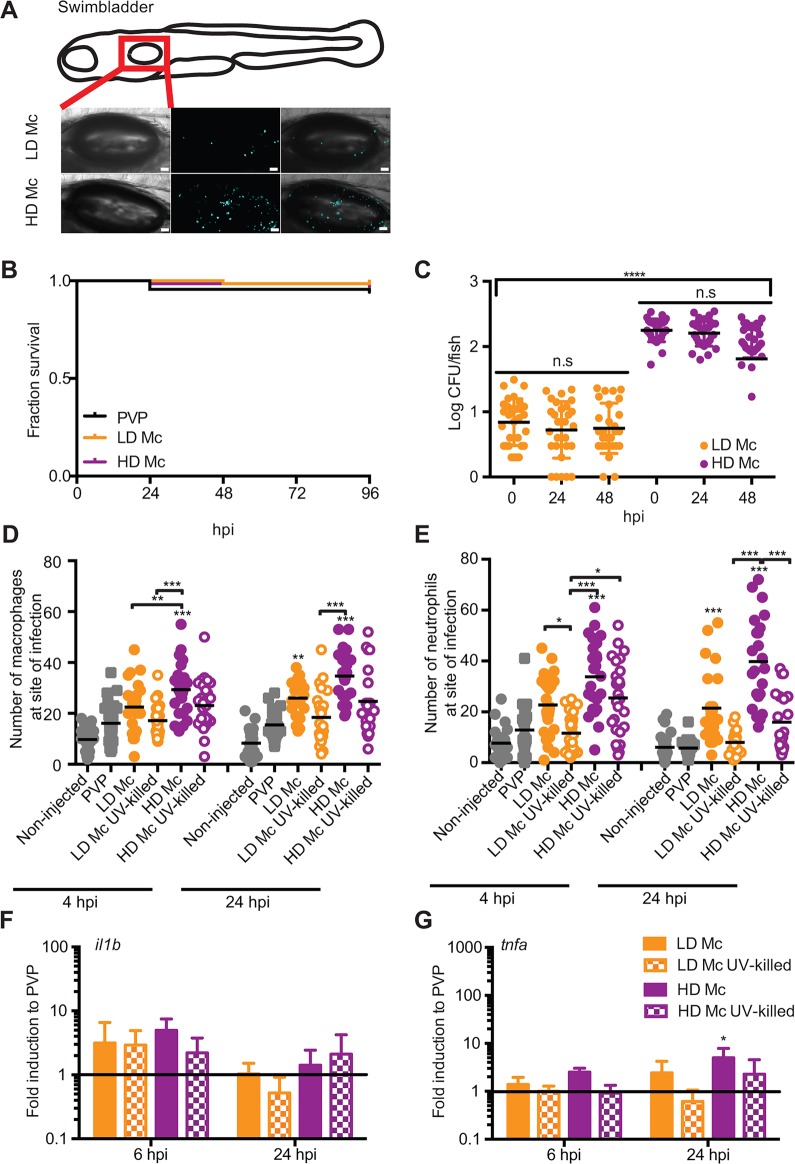
**A zebrafish larval swim bladder infection model for mucormycosis.** (A) AB wild-type zebrafish larvae were injected in the swim bladder 4 days post-fertilization (d.p.f.) with a LD or a HD of *M.* *circinelloides* NRRL3631. (B) No significant mortality was observed after mucormycete spore injection into the swim bladder 4 d.p.f. (PVP versus LD Mc *P*=0.172; PVP versus HD Mc *P*=0.403; LD Mc versus HD Mc *P*=0.561; *n*=68). (C) Spores remained viable within the zebrafish larvae over the time course of 48 h.p.i. (D,E) Macrophages and neutrophils are recruited to the site of infection after injection of viable spores into the swim bladder. Injection of UV-killed spores did not induce significant phagocyte recruitment. (F,G) No significant induction of the pro-inflammatory cytokines *il1b* or *tnfα* was detected at 6 or 24 h.p.i. with the exception of weak *tnfα* induction at 24 h.p.i.

The initial sites of host-pathogen interactions after encounter of mucormycete spores are the mucosal layers lining the nose, lungs and digestive tract. We therefore also investigated a swim bladder infection model, which has previously been applied to investigate mucosal infections with *Candida* *albicans* (Gratacap et al., 2014). Infections in the swim bladder were conducted at 4 days post-fertilization (d.p.f.) with the clinical isolate *M. circinelloides* NRRL3631 at low and high injection dose (10^7^ and 10^8^ spores/ml; Fig. 5A). We would like to highlight that the swim bladder larval infection model is feasible only at a later larval developmental stage (at which point the hindbrain compartment is no longer accessible); hence, it does not allow for direct comparison between the two different sites of infection discussed in this work.

No significant killing was observed in the swim bladder model (PVP versus LD Mc *P*=0.172; PVP versus HD Mc *P*=0.403; LD Mc versus HD Mc *P*=0.561, *n*=68; Fig. 5B) whilst spores remained viable (Fig. 5C). Increased susceptibility was observed in dexamethasone-treated zebrafish larvae after spore injection in the swim bladder (LD Mc DMSO versus LD Mc dexamethasone *P*<0.001; HD Mc DMSO versus HD Mc dexamethasone *P*<0.001, *n*=72; Table 2A). Dexamethasone did not significantly affect larval viability up to 72 h.p.i. (PVP DMSO versus PVP dexamethasone *P*=0.100; Table 2A). Metronidazole did not render larvae more susceptible to fungal spores at the low infection dose in the swim bladder infection model (LD Mc versus LD Mc metronidazole *P*=0.094, *n*=51) and marginally more susceptible at the high infection dose (HD Mc versus HD Mc metronidazole *P*=0.002; Table 2B) within 48 h.p.i. We noted that larval mortality appeared similar between metronidazole-treated larvae after low- and high-dose infection, and significant mortality after the low dose might become apparent after longer observation if the experimental set-up would allow for longer observation. Metronidazole did not alter larval survival up to 48 h.p.i. in the swim bladder infection model (PVP versus PVP metronidazole *P*=0.317; Table 2B). After 48 h.p.i., metronidazole treatment caused significant larval mortality in the PVP control. Taken together, NRRL3631 spores are not virulent in the swim bladder infection model. General immunosuppression with dexamethasone significantly increases larval mortality after spore challenge. Interestingly, macrophage ablation in the swim bladder infection model showed only a weak increase in susceptibility, throwing doubt on the importance of macrophages in protection from fungal spores in this infection model.

**Table 2.**
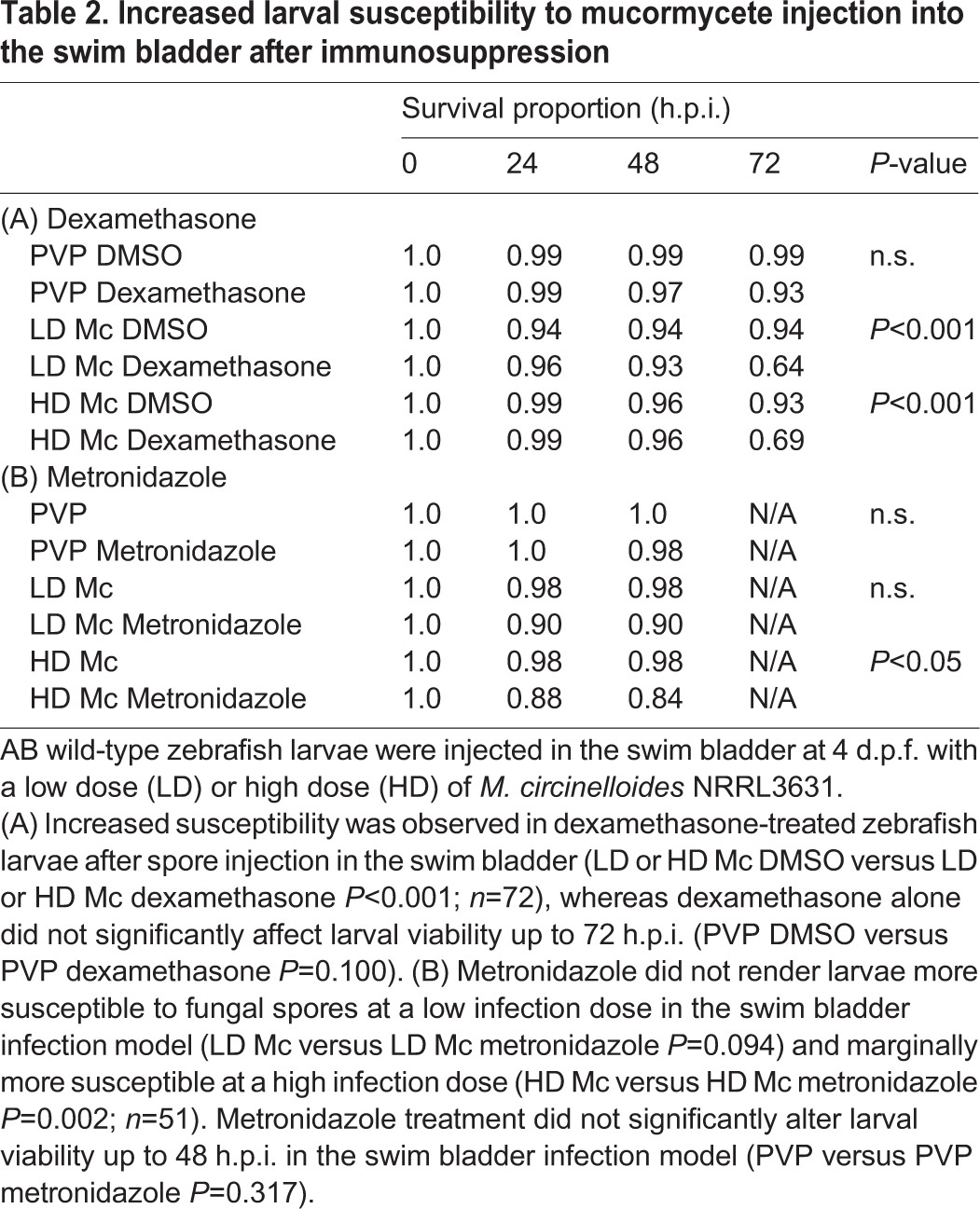
**Increased larval susceptibility to mucormycete injection into the swim bladder after immunosuppression**

Nonetheless, significantly higher numbers of macrophages were found at the site of infection 4 h after injection of a high dose of viable spores compared with the PVP control injections (*P*<0.001) in the swim bladder. No significant macrophage recruitment was seen at this time after injection of low-dose viable or non-viable spores and high-dose non-viable spores. Macrophage recruitment after high-dose challenge with viable spores was also significantly higher than after low-dose challenge with viable spores (*P*<0.01; Fig. 5D and Table S1A). At 24 h.p.i., significantly higher numbers of macrophages were observed in the swim bladder in larvae injected with low- and high-dose viable spores compared with PVP control injections (*P*<0.01 and *P*<0.001, respectively). Injection of non-viable spores resulted in less macrophage recruitment than injection with viable spores at low- and high-dose challenge, although no statistically significant difference was detected at 4 and 24 h.p.i. (Fig. 5D and Table S1A). Significantly higher numbers of neutrophils were found at the site of infection 4 h after injection of a high dose of viable spores compared with the PVP control injections (*P*<0.001) in the swim bladder. No significant neutrophil recruitment was seen at this time after injection of a low dose of viable or non-viable spores and a high dose of non-viable spores. Injection of non-viable spores resulted in less neutrophil recruitment than injection with viable spores after low- and high-dose challenge (LD Mc viable versus non-viable *P*<0.05; HD Mc viable versus non-viable not significant; Fig. 5E and Table S1B). At 24 h.p.i., neutrophil recruitment after low- and high-dose challenge with viable spores was significantly higher than after PVP control injection (*P*<0.001). Injection of non-viable spores showed less neutrophil recruitment than injection with viable spores after low- and high-dose challenge (LD Mc viable versus non-viable not significant; HD Mc viable versus non-viable *P*<0.001; Fig. 5E and Table S1B).

In summary, in contrast to previous *in vitro* studies, macrophages as well as neutrophils are recruited to the site of viable but not UV-killed spore infection in the swim bladder zebrafish larval model of mucormycosis at 4 and 24 h.p.i. UV-killed spores showed reduced neutrophil and macrophage recruitment in swim bladder injections after low- and high-dose challenge. This suggests that both innate immune cells respond in a different manner to swim bladder infection with live versus dead pathogen over time and indicates that differential activation and chemotactic recruitment mechanisms are active during phagocyte recruitment.

No significant levels of cytokine mRNA of the pro-inflammatory mediators *il1b* and *tnfa* were induced by viable or non-viable spores in the swim bladder infection model at either time point, except a small but statistically significant induction for *tnfa* at 24 h.p.i. after high-dose injections of viable spores (Fig. 5F,G). Hence, mucormycete spores elicit a very weak pro-inflammatory cytokine response in the zebrafish larval swim bladder model of mucormycosis.

### *M. circinelloides* virulence can be assessed in immunocompetent larvae

**Fig. 6.**
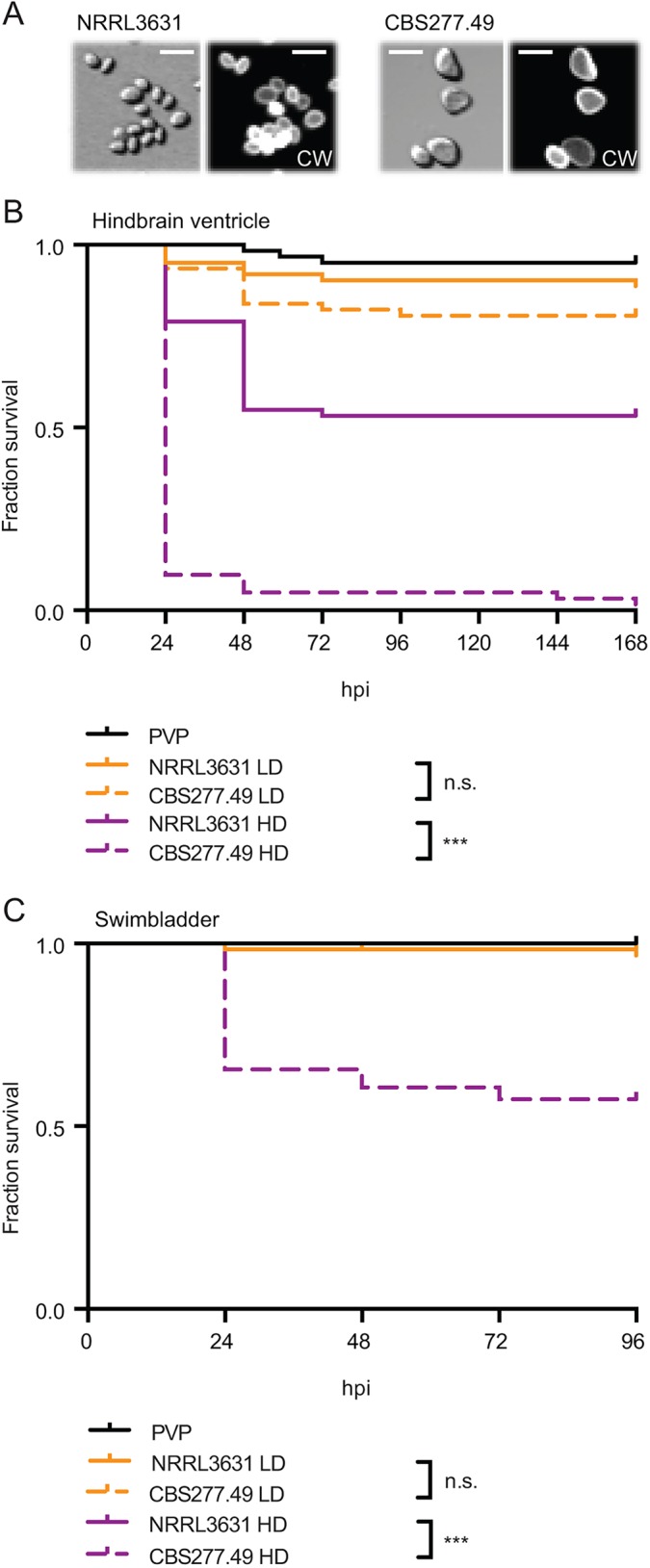
***M. circinelloides* virulence can be assessed in immunocompetent zebrafish larvae.** (A) Large-spore isolate *M. circinelloides* CBS277.49 in comparison to the small-spore isolate *M.* *circinelloides* NRRL3631. CW, Calcofluor White; scale bars: 10 μm. (B) CBS277.49 at a high but not a low infection dose is significantly more virulent compared with NRRL3631 in AB wild-type zebrafish larvae when injected into the hindbrain ventricle. (C) CBS277.49 at a high but not a low infection dose is significantly more virulent compared with NRRL3631 in AB wild-type zebrafish larvae when injected into the swim bladder.

It has been reported that *M. circinelloides* virulence is dependent on spore size, with commonly found smaller spore isolates being less virulent than rarely found isolates with larger spores (Li et al., 2011). We investigated the virulence of a large-spore isolate, *M. circinelloides* CBS277.49, in comparison to the small spore isolate, *M. circinelloides* NRRL3631 (Fig. 6A), in our hindbrain ventricle zebrafish infection model.

Differences in virulence were detected at high infection doses after spore injection into the hindbrain ventricle of immunocompetent larvae (NRRL3631 HD versus CBS277.49 HD *P*<0.001, *n*=62; Fig. 6B) as well as into the swim bladder (NRRL3631 HD versus CBS277.49 HD *P*<0.001; *n*=61; Fig. 6C). At low-dose infection, no differential virulence was detected after spore injection in the hindbrain ventricle (NRRL3631 LD versus CBS477.49 LD *P*=0.217, *n*=62) or swim bladder (NRRL3631 LD versus CBS477.49 LD *P*=0.561, *n*=61) (Fig. 6B,C).

This comparison of mortality after injection with known low- and high-virulence strains demonstrated that both the hindbrain ventricle and the swim bladder infection models are suitable to evaluate mucormycete virulence comparatively at high infection doses.

## DISCUSSION

Here, we report the first real-time *in vivo* analysis of the early innate immune response to mucormycete infection. We have successfully established a new model system, the zebrafish larva, to study host-pathogen interactions during mucormycosis. We applied this whole-animal system to dissect the early host-pathogen events during infections. We identified differential host susceptibility, dependent on the site of infection, together with differential functions of the two major phagocyte effector cell types, the macrophages and neutrophils. Whilst exploring the dynamics of phagocyte recruitment to the infection site, we discovered that clusters are formed in response to fungal spores that potentially exhibit a role in fungal spore dissemination.

We investigated two sites of infection, the hindbrain ventricle, mimicking invasive disease, and the swim bladder, mimicking infection at a mucosal layer (e.g. lung, nose; Brothers et al., 2011, 2013; Gratacap et al., 2013). Invasive infection with extensive hyphal growth occurred in a dose-dependent manner after spore injection into the hindbrain ventricle at prim-25 but not after injection into the swim bladder at 4 d.p.f. This is an interesting observation indicating either tissue-specific susceptibility or differential immune responses in these two sites of infection or, alternatively, developmental differences rendering younger larvae more susceptible. Interestingly, in a study of 929 mucormycosis cases in humans, 62-98% of rhinocerebral, cerebral or disseminated disease resulted in death, whereas localized infection of mucosal layers, such as in the sinuses, was lethal in only 16% of individuals (Roden et al., 2005).

Intranasal challenge of diabetic mice with mucormycete spores results in specific enhanced susceptibility similar to humans with diabetes (Waldorf and Diamond, 1984; Waldorf et al., 1984b). Healthy mice survived infection with spores predominantly retained in the lungs, whereas diabetic mice showed a tropism of high fungal spore burden towards the lung and the brain. This indicates a defect in the specific host immune response to mucormycosis (Waldorf and Diamond, 1984; Waldorf et al., 1984b). However, the specific component responsible for this susceptibility is currently unknown. Although diabetes is one of the hallmark underlying conditions for mucormycosis, corticosteroid treatment, neutropenia, malignancies and traumatic injury also predispose to mucormycete infection (Dromer and McGinnis, 2003; Mendoza et al., 2014). A common feature of all these predisposing factors is that they can result in impaired phagocytic effector cell functions (Mendoza et al., 2014). We induced general immunosuppression in zebrafish larvae after infection with spores by treatment with the corticosteroid dexamethasone and, similar to human mucormycosis, this significantly increased larval mortality. Specific depletion of macrophages in *Tg(mpeg1:G/U:NfsB-mCherry)* larvae with the prodrug metronidazole confirmed previous *in vitro* observations. Macrophages were an essential component of the immune response to mucormycete infection, and macrophage-depleted larvae showed significantly higher mortality than controls in the hindbrain ventricle infection model. Interestingly, a much milder effect was observed in larvae depleted of macrophages after spore injection into the swim bladder, suggesting a less crucial role of this cell type in the containment of the infection in this mucosal model. As both macrophages and neutrophils are recruited to the swim bladder after infection, it is possible that neutrophils play an important role in mucosal defense against mucormycete infection. Neutrophils are known to exhibit a stronger oxidative burst than macrophages (VanderVen et al., 2009). A dominant role of neutrophils and more efficient response to mucormycete spores in the swim bladder might also explain the overall lower mortality levels observed in this infection model.

Previous research has shown differential phagocyte responses to resting or swollen spores and hyphal structures. Although phagocytes can damage fungal hyphae, resting spores (the infectious form) cannot be killed by macrophages nor do they exhibit chemotactic potential on neutrophils (Waldorf et al., 1984a; Waldorf and Diamond, 1985; Levitz et al., 1986; Waldorf, 1989; Jorens et al., 1995). We were able to show macrophage and neutrophil recruitment in our zebrafish larval model. Alveolar macrophages have previously been shown to be required for neutrophil chemotaxis during murine *Pseudomonas aeruginosa* and *Klebsiella pneumonia* infections (Hashimoto et al., 1996; Broug-Holub et al., 1997). Macrophages might also be required for neutrophil recruitment post-infection with mucormycete spores and therefore cannot be observed in two-dimensional *in vitro* interaction experiments. These interactions could potentially be reconstituted in complex *in vitro* systems. However, our zebrafish larval model offers an easily accessible system for direct observation of multidimensional real-time cell-cell dynamics *in vivo*. In the future, this will enable investigation of any immune-manipulatory effects of mucormycete spores on the innate immune system. For example, there is strong evidence for an immune-inhibitory effect of infectious fungal spores on the innate immune system (Levitz et al., 1986; Waldorf, 1989). We investigated the ability of viable and non-viable mucormycete spores to induce expression of the pro-inflammatory cytokines *il1b* and *tnfa*. Pro-inflammatory cytokine mRNA levels were significantly increased only 24 h.p.i. after injection of viable spores into the hindbrain ventricle. Interestingly, non-viable spores did not induce mRNA synthesis of the pro-inflammatory cytokines *il1b* and *tnfa* in the hindbrain, and spore injection into the swim bladder elicited only a weak *tnfa* response.

However, we also observed that fungal spores remained viable within the host without apparent disease up to 48 h.p.i. Bronchoalveolar macrophages have been reported to exhibit inhibitory effects on mucormycete spore germination but fail to kill spores (Waldorf et al., 1984a; Levitz et al., 1986; Waldorf, 1989; Jorens et al., 1995). Histological snapshot studies of intravenous spore injections in rabbits demonstrated an accumulation of leucocytes at the site of infection (Sheldon and Bauer, 1959), and bronchoalveolar lavage after intranasal spore inoculation showed a reduced leucocyte-spore association in diabetic mice (Waldorf et al., 1984b). However, previous *in vitro* research reported a lack of chemotactic potential to induce neutrophil migration in resting spores (Waldorf and Diamond, 1985). We were able to demonstrate macrophage and neutrophil recruitment to both sites of infection in our zebrafish larval model. Interestingly, we repeatedly observed the formation of large clusters of phagocytes and spores at the site of infection with viable spores but not UV-killed spores. Similar structures are well described in tuberculosis, where formation of the host-protective granuloma contains bacteria at the site of infection and restricts bacterial dissemination whilst also acting as a reservoir for later disease reactivation (Ulrichs and Kaufmann, 2006). However, the characteristics of these clusters in the context of *M. circinelloides* infections need to be studied in more detail to determine whether other granuloma-specific attributes are fulfilled (e.g. necrotic tissue, fibroblast and lymphocyte recruitment). Our results so far indicate that cluster formation is correlated with spores being retained at the site of infection. Likewise, zebrafish larval mortality is also correlated with reduced phagocyte recruitment after *Shigella flexneri* infection (Brothers et al., 2013; Mostowy et al., 2013). Therefore, future investigations following macrophage and neutrophil recruitment to the site of infection might explain differences in susceptibility, stratified at individual and tissue levels. Our zebrafish larval model offers the possibility to investigate tissue-specific responses by targeted microinjection into the hindbrain to mimic central nervous system infection (Brothers et al., 2011) or swim bladder to mimic mucosal interactions (Gratacap et al., 2013) similar to those in the pulmonary tract. *In vivo* real-time high-resolution microscopy of the transparent zebrafish larvae will enable the direct investigation of phagocyte recruitment in response to fungal spores in relation to the fate of individual larvae.

Traditional research model systems have been unsatisfactory in deciphering the mechanisms of host phagocyte interaction with fungal spores, besides there being clear evidence for an essential role of innate immunity in an efficient immune response to mucormycosis. We present a valuable whole-animal zebrafish larva model system for *in vivo* high-resolution dissection of mucormycete interactions with the innate immune system. Similar to the human host, our initial investigations highlighted the importance of phagocytes in mounting a protective immune response to fungal spores as well as several aspects of differential responses to infection with mucormycete spores in the zebrafish model. The zebrafish immune system is very similar, at the molecular and cellular levels, to the human equivalent. In addition, the availability of whole-genome data and thousands of mutant lines of this easily and cheaply bred whole-animal system offers huge advantages for advancing our understanding of mucormycosis. In the future, this model system will enable the direct study of the effect of well-established host determinants on phagocyte function and disease progression to explain increased host susceptibility. Zebrafish larvae can be rendered diabetic by targeted ablation of β-cells (Pisharath et al., 2007), iron overdose can be induced by growth in high-iron-containing media or treatment with deferoxamine (Chamilos et al., 2008b), and immunosuppression can be induced by treatment with dexamethasone. Excitingly, the transparent larvae will allow comparative microscopic real-time *in vivo* investigations of phagocyte recruitment, phagocyte accumulation and spore dissemination in the susceptible versus healthy host.

Further research into the interactions between innate immune effectors and mucormycete spores using this *in vivo* model system will significantly improve our understanding of the innate immune response during mucormycosis and therefore potentially contribute to the development of new therapeutic strategies for this pathogen that is very difficult to manage clinically.

## MATERIALS AND METHODS

### *M. circinelloides* strains and growth conditions

This study used the two published and characterized *M. circinelloides* strains NRRL3631 [(+)-mating type] and CBS277.49 [(−)-mating type] (Li et al., 2011) that produce small, less virulent and large, highly virulent spores, respectively. Strains were grown and maintained on Sabouraud dextrose agar pH 5.6 containing 1% (w/v) mycological peptone, 4% (w/v) dextrose and 1.5% (w/v) agar (Merck, Kenilworth, NJ, USA). Spores were plated at a density of 1000 spores per 10 cm agar plate, and plates were kept at room temperature in the dark for 4 days before experimental use.

### Zebrafish care and maintenance

Zebrafish were kept in recirculating systems (Aquatic Habitats, Apopka, FL, USA) at the University of Maine Zebrafish Facility. Zebrafish were kept under a 14 h-10 h light-dark cycle with water temperature maintained at 28°C. All zebrafish care protocols and experiments were performed in accordance with National Institutes of Health (NIH) guidelines under Institutional Animal Care and Use Committee protocol A2009-11-01. After collection of eggs, larvae were kept in an incubator at 33°C at 40 eggs per 50 ml E3 media plus 0.00003% methylene blue for 8 h and E3 media plus 26.6 µg/ml 1-phenyl-2-thiourea (PTU; Sigma-Aldrich, St Louis, MO, USA) thereafter. PTU at this concentration inhibits melanization; hence, it allows microscopic investigation without showing any impact on larval viability and development. This has been reported by others and was routinely monitored during our experimental work by inspection of larval survival and anatomical development up to 8 days post-fertilization (d.p.f.). Medium was changed every 2 days. The fish lines used were wild-type AB zebrafish from Zebrafish International Resource Collection (ZIRC) as well as the transgenic zebrafish *Tg(mpx:GFP)^i114^* expressing green fluorescent protein in neutrophils (Renshaw et al., 2006), *Tg(mpeg1:Gal4-FF)^gl25^* (Ellett et al., 2011; a generous gift from G. Lieschke, Monash University) crossed with *Tg(UAS-E1b:NfsB.mCherry)^c264^* [herein referred to as *Tg(mpeg1:G/U:NfsB-mCherry)*] with macrophage-specific expression of red fluorescent protein mCherry and nitroreductase, and *Tg(mpeg1:Gal4-FF)^gl25^/Tg(mpx:GFP)* crossed with *Tg(UAS-E1b:NfsB.mCherry)^c264^* [herein referred to as *Tg(mpeg1:G/U:NfsB-mCherry/mpx:GFP)*] with neutrophil-specific expression of green fluorescent protein and macrophage-specific expression of red fluorescent protein mCherry. All zebrafish care and husbandry procedures were performed as previously described (Westerfield, 2000).

### Spore preparation for injection

Spores were collected with 10 ml of PBS. The spore suspension was washed and spore concentration was counted in a haemocytometer. To visualize spores, 10^6^ spores were stained in 100 µl PBS containing 100 µg/ml Fluorescent Brightener 28 (Sigma-Aldrich) and 0.1 M NaHCO_3_ for 30 min protected from light in a bench-top rotator. Afterwards, spores were washed three times with PBS and the concentration was adjusted to 10^7^ or 10^8^ sporangiospores/ml in 10% (w/v) polyvinylpyrrolidone (40 kDa; Sigma-Aldrich) with 0.05% phenol red in ddH_2_O. Staining did not affect spore ability to germinate (Fig. S2).

For experiments using dead spores, the spores were killed by UV irradiation. Spores suspended in 20 ml PBS were irradiated twice for 15 min in a UVP CL-1000 UV crosslinker with intermediate PBS washing. Spores were collected and stained as described above.

### Hindbrain ventricle infection

Hindbrain infections were conducted as previously described (Brothers et al., 2011). Zebrafish development was assessed according to Kimmel et al. (1995) and injected at prim-25 stage. Zebrafish were manually dechorionated and anaesthetized with 160 µg/ml Tris-buffered tricaine methane sulfonate (Tricaine; Western Chemicals, Frendale, WA, USA). Transgenic zebrafish were screened at prim-25 stage and only larvae expressing the fluorescently labelled cell(s) selected for injection. For injections, 2 nl of PVP (10% in PBS) or *M. circinelloides* suspension at 10^7^ or 10^8^ sporangiospores/ml in PVP was microinjected through the otic vesicle into the hindbrain to achieve a dose of approximately 10 or 100 spores/larva (low and high dose), respectively. PVP is a carrier medium with increased density that prevents fast clogging of injection needles and also increases consistency of inoculum size. Larvae were screened immediately after injection to confirm inoculum location and infection dose; only larvae with the approximate correct inoculum were selected. Larvae were monitored daily thereafter for survival studies up to 8 d.p.f. At 8 d.p.f. the larvae were killed with a 10× overdose of Tricaine.

### Swim bladder infection

Swim bladder injections were conducted as previously described (Gratacap et al., 2014; Gratacap and Wheeler, 2014). Zebrafish were maintained as described above until 4 d.p.f. At 4 d.p.f., larvae were anaesthetized with 160 µg/ml Tricaine, and only larvae with an inflated swim bladder were microinjected in the swim bladder with 2 nl PVP or *M. circinelloides* suspension at 10^7^ or 10^8^ sporangiospores/ml in PVP. Larvae were screened immediately after injection to confirm inoculum location and infection dose and monitored daily thereafter for survival studies up to 8 d.p.f. At 8 d.p.f., larvae were killed with a 10× overdose of Tricaine.

### Spore viability assessment

To test spore survival, individual larvae (a total of 10 per condition/experimental repeat) were killed with a 10× overdose of Tricaine, homogenized in 100 µl penicillin-streptomycin (5000 U/ml-5 mg/ml; Lonza, Anaheim, CA, USA) and gentamicin (10 mg/ml; Lonza) (5:1) using pellet pestles and plated onto YPD agar (1% BactoYeast, 2% BactoPeptone, 2% Dextrose and 2% agar) with 100 U/ml-100 µg/ml penicillin-streptomycin and 30 µg/ml gentamicin. Plates were incubated at room temperature for between 24 and 48 h, and CFU were counted.

### Fluorescence microscopy

For live imaging, zebrafish larvae were anaesthetized with 200 µg/ml Tricaine and immobilized in 0.4% low-melting point agarose (Lonza) in E3 in a 24-well plate glass-bottomed imaging dish (MatTek, Ashland, MA, USA). Screening after injection and phagocyte recruitment analysis (at 4 and 24 h.p.i.) were performed with a Zeiss Axiobserver Z1 microscope equipped with a VivaTome system (Carl Zeiss Mircoimaging, Melville, NY, USA). Confocal microscopy was performed with an Olympus IX-81 inverted microscope with an FV-1000 laser scanning confocal system (Olympus, Waltham, MA, USA). Objective lenses with powers of 4×/0.16 numerical aperture (NA), 10×/0.4 NA, 20×/0.7 NA and 40×/0.16 NA (Olympus, USA) were used. Enhanced green fluorescent protein, mCherry and Fluorescent Brightener fluorescent signals were detected by excitation at 488, 543 and 405 nm, respectively. Images were collected and processed using Fluoview imaging software (Olympus) and Photoshop (Adobe Systems Inc., San Jose, CA, USA). Time-lapse imaging was performed over 12 h time periods, with *Z*-stack images taken every 5 min and movies generated with ImageJ. Figures represent single-slice differential interference contrast channel (DIC) and maximal projection of fluorescent image channels (UV, green, red) either independently or overlaid. The number of slices for each maximal projection is specified in individual figure legends.

### Chemical treatments

General immune suppression was induced in AB wild-type larvae by treatment with the steroid drug dexamethasone (Calbiochem, San Diego, CA, USA). Larvae were bathed with low-dose dexamethasone in E3 media (10 µg/ml) or equivalent control dose (0.5% DMSO). Macrophage depletion studies were conducted by bathing of transgenic *Tg(mpeg1:G/U:NfsB-mCherry)* larvae with macrophage-specific expression of *E. coli* nitroreductase and fluorescent marker mCherry (Davison et al., 2007) in metronidazole-containing E3 media. Depletion was achieved by an initial 4 h treatment with 20 mM metronidazole in E3 media prior to injection and maintained after injection by addition of 10 mM to E3 media. Metronidazole is a prodrug that is converted into a cytotoxic DNA cross-linking form in nitroreductase-expressing cells, inducing apoptosis and specific cell ablation (Curado et al., 2007; Davison et al., 2007; Pisharath et al., 2007). Successful depletion was assured by counting of mCherry^+^ cells in the caudal haematopoetic tissue, six somites posterior to the anal vent at 0, 4 and 24 h of treatment (Fig. S1A,B). *Tg(mpeg1:Gal4-VP16)* ﬁsh were crossed with *Tg(UAS-E1b:nfsb-mCherry)* ﬁsh and reared at 33°C for 4 days in E3+PTU. Fish were anaesthetized, imaged by confocal microscopy, and mCherry^+^ cells were counted (*n*=30) in the caudal hematopoietic tissue, six somites posterior to the anal vent. Fish were then divided into two groups (*n*=15 each) and treated with either E3+PTU (0 h post-treatment=0 h.p.t.) or 20 mM metronidazole in E3+PTU (0 h.p.t.). After 4 h of treatment, ﬁsh were imaged by confocal microscopy (4 h.p.t.), and mCherry^+^ cells were counted in −Met and +Met groups (4 h.p.t. −Met and +Met). Fish were then treated with either E3+PTU or 10 mM metronidazole in E3+PTU. Fish were imaged at 24 h.p.t. (24 h.p.t.) and mCherry^+^ cells counted (24 h.p.t. −Met and +Met). Both chemical treatments were commenced after microinjection of fungal spores and terminated as soon as adverse effects resulting from chemical treatment were observed. Zebrafish larvae were monitored daily for survival studies.

### Assessment of phagocyte cluster formation and spore dissemination

To quantify phagocyte cluster formation and spore dissemination, *Tg(mpeg1:G/U:NfsB-mCherry)* and *Tg(mpx:GFP)* zebrafish larvae were injected with a low or a high dose of viable or non-viable spores and observed 24 h.p.i. Positive cluster formation was defined as accumulation of >10 phagocytes in a 40 μm ×40 μm area. A dissemination event was recorded if there was at least one spore that was observed outside the hindbrain ventricle compartment.

### RNA isolation and quantitative real-time PCR

Wild-type zebrafish larvae injected with PVP only or with a low or high dose of spores in the hindbrain ventricle or in the swim bladder were killed with a 10× overdose of Tricaine at 6 and 24 h.p.i., and total RNA was isolated from 15 whole larvae using a combination of TRIzol^®^ (Invitrogen, Waltham, MA, USA) and RNeasy column (Qiagen, Valencia, CA, USA). Initially, the TRIzol^®^ isolation protocol was followed. The aqueous phase containing RNA was transferred to RNeasy columns and RNA samples were cleaned up according to the manufacturer’s instructions. Total RNA was eluted in 20 µl of nuclease-free water and stored at −80°C until further use. cDNA was synthesized from 500 ng of total RNA with the iScript™ cDNA synthesis kit (Bio-Rad). A no-RT reaction was performed for each sample. qPCR reactions were carried out using a CFX96 thermocycler (Bio-Rad, Berkeley, CA, USA) with the primers detailed in Table S2 and the following conditions: 3 min at 95°C, followed by 40 cycles of 5 s at 95°C and 20 s at 58°C; a final dissociation curve between 65 and 95°C was performed for each group. Threshold cycles (Ct) and dissociation curves were analysed with Bio-Rad CFX Manager software. Gene expression levels were normalized to zebrafish *gapdh* (ΔCt) and compared with PVP-injected controls (ΔΔCt). Results are presented as fold induction (2^ΔΔCt^; Gratacap et al., 2013).

### Data analysis and statistics

All data were collected from at least three independent experimental repeats. Exact numbers of experimental repeats and larvae investigated are given in each results section or figure legend. Data were analysed with GraphPad Prism version 6.0 software (GraphPad, La Jolla, CA, USA). Statistical analysis of survival curves (pooled data from three independent experimental repeats) was performed using Mantel-Cox log-rank test. Cytokine responses were analysed by two-way ANOVA with Tukey's multiple comparisons test. Data from CFU counts, macrophage ablation and phagocyte recruitment were compared using the non-parametric Kruskal–Wallis test with Dunn's multiple comparison test. Categorical data for phagocyte clustering and dissemination were compared by two-tailed Fisher's exact test. Values of *P*<0.05 were considered statistically significant and are indicated in figures as *(*P*<0.05), **(*P*<0.01) or ***(*P*<0.001).

### Ethics statement

All zebrafish care protocols and experiments were performed in accordance with NIH guidelines under Institutional Animal Care and Use Committee protocol A2009-11-01.
